# A comparative evaluation of the process of developing and implementing an emergency department HIV testing program

**DOI:** 10.1186/1748-5908-6-30

**Published:** 2011-03-30

**Authors:** Katerina A Christopoulos, Kim Koester, Sheri Weiser, Tim Lane, Janet J Myers, Stephen F Morin

**Affiliations:** 1 San Francisco General Hospital HIV/AIDS Division, University of California San Francisco, San Francisco, CA, USA; 2 Center for AIDS Prevention Studies, University of California San Francisco, San Francisco, CA, USA

## Abstract

**Background:**

The 2006 Centers for Disease Control and Prevention (CDC) HIV testing guidelines recommend screening for HIV infection in all healthcare settings, including the emergency department (ED). In urban areas with a high background prevalence of HIV, the ED has become an increasingly important site for identifying HIV infection. However, this public health policy has been operationalized using different models. We sought to describe the development and implementation of HIV testing programs in three EDs, assess factors shaping the adoption and evolution of specific program elements, and identify barriers and facilitators to testing.

**Methods:**

We performed a qualitative evaluation using in-depth interviews with fifteen 'key informants' involved in the development and implementation of HIV testing in three urban EDs serving sizable racial/ethnic minority and socioeconomically disadvantaged populations. Testing program HIV prevalence ranged from 0.4% to 3.0%.

**Results:**

Three testing models were identified, reflecting differences in the use of existing ED staff to offer and perform the test and disclose results. Factors influencing the adoption of a particular model included: whether program developers were ED providers, HIV providers, or both; whether programs took a targeted or non-targeted approach to patient selection; and the extent to which linkage to care was viewed as the responsibility of the ED. A common barrier was discomfort among ED providers about disclosing a positive HIV test result. Common facilitators were a commitment to underserved populations, the perception that testing was an opportunity to re-engage previously HIV-infected patients in care, and the support and resources offered by the medical setting for HIV-infected patients.

**Conclusions:**

ED HIV testing is occurring under a range of models that emerge from local realities and are tailored to institutional strengths to optimize implementation and overcome provider barriers.

## Background

The 2006 Centers for Disease Control and Prevention (CDC) guidelines recommend routine HIV screening in all healthcare settings where the HIV prevalence exceeds 0.1%, including the emergency department (ED) [1]. In 2007, the American College of Emergency Physicians (ACEP) formally endorsed the mission of HIV testing in EDs, provided that it did not interfere with the provision of emergency care, was in compliance with state laws, and was appropriately funded [2]. In 2009, there were over 20 CDC, public health department, National Institutes of Health (NIH), and industry-funded ED HIV testing programs at academic medical centers across the United States [3].

Though the CDC guidelines endorse non-targeted screening and opt-out consent, ED HIV testing is currently occurring under a range of operational models, with variation in patient selection strategies, methods of consent, test choice, and use of support staff [4]. Lessons learned from implementing these models include the importance of an ED testing 'champion,' early buy-in from key partners, quality control, protocols that address education, disclosure, and linkage to care, feedback to ED clinicians, and mechanisms for funding and sustainability [5,6]. To our knowledge, there has been no qualitative study of the process of developing and implementing an ED HIV testing program. Qualitative methods are useful for understanding the rationale for programmatic choices in public policy implementation and for identifying barriers to and facilitators of the operationalization of policy guidelines. As such, qualitative research can contribute important knowledge to health services evaluation.

In 2008, the CDC provided funding to state and local health departments to implement the 2006 guidelines and increase the number of persons tested for HIV, particularly in populations disproportionately affected by HIV, such as African-Americans. Through this mechanism, the California State Office of AIDS awarded grants to three EDs in northern California to expand or initiate HIV testing. The objectives of this study were to: characterize and compare these ED HIV testing programs in terms of their procedures; assess factors shaping the adoption and evolution of different testing models; and identify site-specific and common barriers to and facilitators of HIV testing.

## Methods

### Study design

This study was a qualitative evaluation of the development and implementation of three ED HIV testing programs using in-depth interviews with key program personnel. The institutional review boards of the University of California San Francisco and all participating sites approved this study.

### Setting

Of the three EDs, two belonged to county hospitals with emergency medicine residency training programs and one was part of a not-for-profit community health system. The average annual number of patient visits at the county EDs was between 60,000 and 75,000, while the community hospital ED had a smaller census at 40,000. All EDs were located in urban areas and served sizable racial/ethnic minority and socioeconomically disadvantaged populations. According to internal data, the prevalence of HIV infection in these ED testing programs ranged from 0.4% to 3.0%.

### Selection of participants

Participation in this study was restricted to individuals who were involved in the development or implementation of ED HIV testing. Recruitment began with meetings of researchers and the principal investigators (PIs) of the CDC grant at each site. At the meetings, researchers described the objectives of the study, invited PI participation, and asked site PIs to identify and help recruit key staff involved in the development and implementation of the ED testing programs. Researchers then contacted participants and set up mutually convenient times for interviews at each site.

Fifteen staff members (five from each program) took part in the study, including ED and HIV clinic physicians, ED and HIV clinic nurses, program coordinators, and staff hired to administer HIV tests, who will be referred to as dedicated testers. This purposeful sample was recruited to understand the perspectives of program developers and implementers at the three sites. These fifteen staff members included all key personnel from the implementation of each program.

### Data collection and processing

Study data were collected from January to May 2009 by four investigators with experience in qualitative research. Researchers conducted in-depth interviews with study participants that lasted 30 to 60 minutes and were recorded and transcribed verbatim. Whenever possible, researchers interviewed participants in teams of two. Each researcher conducted between three and seven interviews. Researchers met to discuss their findings on an ongoing basis.

A semi-structured interview guide was developed to elucidate perspectives on the motivations and challenges of providing HIV testing in the ED. The interview questions were situated within the broader context of federal and state policies designed to encourage HIV testing as part of routine medical care. Specifically, the interview guide covered five areas: the participant's role and level of involvement in program development and implementation; a description of program procedures, with specific attention to the testing process elements outlined by the National ED HIV Testing Consortium [7], including patient selection, consent, testing methods, pre-result communication, post-result communication, and outcome measures, with an emphasis on linkage to care; the program planning and implementation process; facilitators and barriers to ED HIV testing, including possible solutions to barriers; and the participant's thoughts on whether screening for HIV should be the same as for other chronic diseases, such as diabetes or hypertension. Although the interviews covered each of these five topic areas whenever possible, they were also iterative in nature, in that the interviewers followed up on spontaneously offered information and raised issues they had learned about in previous interviews. Each site received $250 for participating in the study.

### Primary data analysis

Three members of the research team collaborated on data analysis. All had been involved in data collection, which contributed to an in-depth understanding of the structure of each program as well as of the roles of the staff participating in the research. These team members represented different disciplines, including medicine/health services research, medicine/anthropology, and anthropology/health services research. Framework analysis was used to analyze the data. This type of analysis originated in the context of applied social policy research and its benefits include five transparent stages of analysis that follow a well-defined process [8]. First, the researchers familiarized themselves with the individual interviews, noting key content areas (familiarization). Next, the researchers met as a group to identify salient coding categories to be applied across interviews (identifying a thematic framework). The codebook consisted of both *a priori *codes derived from the evaluation objectives to characterize and compare the HIV testing models along with barriers and facilitators as well as emergent codes. The research team applied the codes and convened analysis retreat meetings to read and summarize the data as a group (indexing). Together the team created tables of the data associated with the codes deemed most relevant (charting). The final phase involved comparing and contrasting the models to understand the similarities and differences of each case (interpretation).

## Results

### ED HIV testing models

We identified three distinct ED HIV testing models. The central difference between the models was the extent to which existing staff were used to carry out the activities associated with HIV testing, including offering the test, performing the test, and disclosing results. The first model hired dedicated testers to accomplish HIV testing. A second model relied fully on existing staff: physicians offered the HIV test and the hospital laboratory performed it. A third model combined elements of the first two models, where triage nurses offered the HIV test, dedicated testers conducted it, and physicians disclosed all results. For convenience, the first model will be referred to as the parallel model, the second model will be called the provider model, and the third model will be known as the provider-parallel model. See Table 1 for a comparison of testing models.

**Table 1:** Characteristics of three emergency department HIV testing programs

Testing Model	Testing Program Planners	Program Already Existed at CDC Grant Award?	Rapid Test Type	Patient Selection Criteria	Test Offer and Consent	Pre-test Counselling	Test Performer	Test Results and Confirmation	Disclosure	Linkage to Care
Parallel	ED clinicians	Yes	Oral swab	Non-targeted	Signed opt-out at registration	At the discretion of the tester	Tester, almost 24/7	Rapid test results in 20 minutes Blood drawn in ED for confirmation	Tester discloses negative results; physician discloses positive results	Referral to guaranteed HIV clinic drop-in appointment

Provider	HIV clinicians and ED clinicians	Yes	Venipuncture specimen	Targeted to all admitted patients and symptoms/risk factors	Verbal opt-in by physician; implied consent if impacts care	At the discretion of the physician	Hospital laboratory 24/7	Results available in electronic medical record in 1 to 2 hours Confirmation done on same specimen	Physician discloses negative and positive results	Dedicated HIV clinic-based linkage to care team who will meet patient at disclosure

Provider -Parallel	HIV clinicians and ED clinicians	No	Oral swab	Non-targeted	Verbal opt-in by triage nurse	None	Tester, almost 24/7	Blood drawn in ED for confirmation	Physicians disclose negative and positive results	Dedicated linkage to care liaison who will meet patient at disclosure

All programs studied used rapid antibody testing. However, the programs with parallel staff conducted oral fluid swabs at the point of care, while the provider model used the hospital laboratory to perform rapid testing on blood drawn by nurses. Point of care testing gave results in 20 minutes while the hospital laboratory reported results in the electronic medical record one to two hours after specimen receipt. The hospital laboratory offered HIV testing around the clock, while the parallel models had the staff capacity to test for most, but not all, of a twenty-four hour period. Negative rapid test results were considered HIV-negative results. Positive oral swabs were confirmed with blood drawn in the ED, while positive rapid tests on the venipuncture specimens used in the provider model were confirmed using the same specimen.

### Factors shaping the adoption and evolution of different testing models

In comparing program implementation, we found that three related factors appeared to influence the adoption of a particular testing model: whether programs took a targeted or non-targeted approach towards patient selection for testing; whether program developers were ED providers, HIV primary care providers, or both; and the extent to which developers viewed linkage to care as a primary responsibility of the ED testing program rather than that of HIV providers. Programs that took a non-targeted approach to testing - *i.e*., the parallel and provider-parallel models - relied on support staff to offer and perform testing, while the provider model utilized a targeted approach, choosing to incorporate testing into the duties of existing staff. Programs where the HIV clinic was actively involved in development and implementation of the ED testing program had integrated linkage to care mechanisms in which staff usually came to the ED to meet patients with positive rapid test results.

### Site one: The parallel model

The desire to provider better patient care through diagnostic HIV testing initially motivated this ED to begin an HIV testing program. As stated by one ED physician:

'It had nothing to do with HIV screening at all. I'm an emergency medicine physician and I like to make diagnoses and I got frustrated because I would see patients that I was convinced had an AIDS-defining illness or clinical stigmata of AIDS and I couldn't make a definitive diagnosis in the emergency department.'

With the ability to perform diagnostic testing, the program became more committed to screening, in part because of the scope of available grants. This program initially used existing staff (in this case, ED nurses) to offer and perform non-targeted, point of care testing and shifted to parallel staff only after some limitations to using ED providers to screen patients became evident. One ED physician stated:

'It was always a struggle getting them to routinely offer and it was a struggle for a number of reasons. One is that they had a hard time figuring out who was eligible and who was ineligible. 'This patient looks a little too sick.' They would definitely profile who they would ask, so we didn't get true screening by having the nurses do it. They would definitely look at a little old lady and probably not offer it to them.'

Additional barriers were that nurses were busy, with competing time demands, and some simply felt uncomfortable offering patients an HIV test. An important logistical barrier was that patients who agreed to test did not always complete the test because of a lack of available staff to actually perform the test.

Having shown that HIV testing in the ED was feasible, program developers felt comfortable expanding screening by hiring dedicated testers. The program moved the offer of testing to ED registration, where clerks asked patients to sign a box if they did not want to be tested for HIV (opt-out screening). The primary barrier identified under this organizational model was the staffing challenge of maintaining quality control of the point of care testing system according to laboratory standards.

Though this program identified the hospital HIV clinic as a key partner in initiating HIV testing, the HIV clinic was not actively involved in the testing program, and linkage to care in both phases of the program consisted of referral by the ED to a guaranteed drop-in appointment at the clinic. As one ED physician described:

'Our job as the ED is to disclose, make sure the confirmatory testing gets done, and then give them follow up in one of our clinics. And then the docs are done and it becomes the responsibility of the clinic to get them in....'

This view of linkage to care was situated within a larger understanding of the role of the ED in triaging and treating patients:

'Our goal is for patients to earn their preliminary result, be able to answer questions, and get them to the next step. That is kind of like the model of emergency medicine physicianship: where does this patient need to be, either I can fix it today or I need to get them to the right hands....'

### Site two: The provider model

In contrast to the screening and referral program at the first site, the second site implemented a targeted testing program with an integrated linkage component that built upon existing systems in the hospital laboratory and HIV clinic. Indeed, the ED was part of a larger hospital-wide effort to change the hospital HIV testing platform from batched enzyme immunoassay testing with results available every two to three days to rapid antibody testing. Providers from the HIV clinic were actively involved in creating the ED testing program and expanding a pre-existing clinic linkage to care program to the ED. Prior experience with a successful domestic violence screening program within the ED also helped shape the approach of the HIV testing program. As explained by one of the ED physicians:

'So the first phase was to offer it, do some education, see if we could link patients to care. See if the lab could handle it, see if the physicians could handle it, see if we were going to lose patients or not. So test the system small....It can be small and modest but it needs to work. I don't want to introduce a big, overwhelmingly ambitious program that fails. And we did the same thing with domestic violence.'

Providers were encouraged to test patients with symptoms and signs of HIV infection. Program developers described rejecting a suggestion from potential funders to consider changing their model of testing, as they believed that targeted testing with an emphasis on linkage to care was more important than screening widely. One HIV clinic nurse described the rationale as follows:

'We were told we would like you to expand this thing and go to what the model has been in other EDs which is to do screening and hire test counsellors and all that and we said no, that we didn't want to; that we really thought we were onto something in terms of the model; that for us the critical priority was - the reason we were wanting to test people was to link them into care and it wasn't to test them for testing purposes to get the results.'

With CDC funding, the program expanded its testing criteria to include all admitted patients and patients with risk factors for HIV infection. This staff member went on to emphasize the effort involved in successful linkage to care:

'And the linkage to care piece is really, really intensive; it's not just appointment reminders and stuff it's meeting people in the emergency department, helping with disclosure, immediate test counselling, post-disclosure counselling, partner notification, some general education and then making the appropriate links here at the clinic end matching the patient with an appropriate provider, ensuring the transfer of medical information from their inpatient stay and their diagnosis to the new medical provider giving them rapid and easy access to the clinical services. Also being available for the confirmatory test for non-admitted patients so for patients who are seen in the ED and get their HIV test done there.'

The main barrier identified at this site was resistance on the part of ED physicians to offer what they viewed as a test to be performed in a primary care setting. Program implementers described how buy-in on the part of ED physicians increased once they experienced how the ability to diagnose HIV in the ED could directly impact and improve the management of the patient's presenting ED complaint.

### Site three: The provider-parallel model

At this site, the HIV clinic approached the ED about obtaining CDC grant funding to implement a testing program using site one as a model. ED staff were receptive to this proposal because they felt their patient population was similar in terms of demographics to site one and could benefit from this type of initiative. One ED nurse recalled: 'And I thought that this program was an excellent program; something really good for the community.' In creating their program, staff at this ED chose to adopt some, but not all, parts of the original site one model. Similar to site one, triage nurses offered patients the test. However, dedicated testers were hired to carry out point of care testing and physicians disclosed both negative and positive test results. They also created a position for an HIV clinic-based linkage to care staff person to act as a liaison between the ED and the clinic. Similar to site two, this staff member met patients in the ED at the time of diagnosis to help conduct confirmatory testing and provide education and support. As at site one, this program found that a key barrier to implementation was discomfort on the part of some triage nurses with offering patients the test. In addition, some nurses felt that certain HIV testing informational materials were too graphic. The primary logistical barrier at this site was ensuring that physicians remembered to give negative test results to patients.

### The common barrier to ED HIV testing: Concern about disclosing a positive HIV result

Many of the attitudinal and logistical barriers to HIV testing in the ED were site-specific and depended on the details of the particular model of testing. Attitudinal barriers included discomfort with offering the test, discomfort with HIV informational materials, viewing HIV testing as within the domain of primary care, and 'profiling' patients as appropriate or not appropriate for an HIV test. Logistical barriers included competing time demands, lack of staff to offer and perform testing, remembering to disclose negative test results, and quality control for point of care testing (Table 2). However, across all models, ED physicians were responsible for disclosing a positive HIV result and program developers described some concern and anxiety on the part of ED physicians with regard to this disclosure. One HIV physician described the response of an ED physician:

**Table 2:** Barriers and facilitators to ED HIV testing

Site-Specific Barriers	Common Barrier
'Profiling' patients as appropriate or not appropriate for an HIV test	Discomfort about disclosing a positive HIV test result
Discomfort about offering an HIV test	
Discomfort about HIV informational materials	
Viewing the HIV test as within the domain of primary care	**Common Facilitators**
Competing time demands	Serving vulnerable urban populations
Lack of staff to offer and perform the test	The secondary gain of re-engaging known HIV-infected patients back into care
Quality control for point of care testing	The support of the medical setting, *e.g*., social services, medical evaluation.
Remembering to disclose negative test results	

'I can't tell somebody they have HIV. That's too devastating....Yeah, I don't have a problem telling a family that their six-year-old was killed in a car accident. I can do that. But I can't tell someone they have HIV.'

One linkage to care staff member felt that this discomfort was due in part to a stigmatized view of HIV:

'We're convinced that one of the reasons that the clinicians say we can't disclose a positive HIV test result in the emergency department really comes from stigma. Because they disclose bad news all the time in emergency departments. You know, 'Mrs. Jones, we're really sorry to tell you your son was shot and died,' or, 'Mrs. Jones, you're 35-years-old and you came in for back pain and guess what: you have metastatic breast cancer.''

This staff member observed that ED HIV testing initiatives could play an important role in normalizing perceptions of HIV among healthcare providers, not just patients. To address concerns about disclosure, programs provided educational sessions about HIV along with disclosure scripts and simple confirmatory testing algorithms.

### Common facilitators of HIV testing in the ED

#### Serving vulnerable urban populations

Program developers at all three sites framed HIV testing as a way to improve care for underserved populations. Many staff noted that individuals who were poor, unstably housed, or from racial/ethnic minorities often did not typically access medical care elsewhere. One HIV clinic nurse observed:

'...there are a lot of patients who don't know their HIV status and many of them are patients that don't have primary care providers and only access healthcare through the emergency department and urgent care. So it was, you know, an extension of really trying to link marginalized populations who don't have access to HIV care, link them into care and so it was sort of a natural, logical extension to get into testing and put testing in the equation.'

An ED physician expressed a moral imperative to provide this service:

'The response was overwhelmingly supportive, like this is absolutely something we should do. So they were buying into it on this emotional civic duty. We work at a county hospital. We owe it to our patients. They have nowhere else to go.'

#### The secondary gain of re-engaging known HIV-infected patients in care

Programs also alluded to the 'secondary gain' of being able to connect HIV-infected patients who were not in care back into care. They observed that some patients accepted the offer of testing without disclosing their positive status and that this re-testing provided the opportunity for re-entry into care.

'If they say 'I already have HIV' then I always tell them make sure you ask 'Are they in care?' [Be]cause there are some that are not and they will give them my card and I'll contact them. Just a week ago I had somebody who was in the ER for ETOH, which is intoxicated. And he got tested; he didn't tell us that he was already positive. He's been positive for 15 years. When we were done the nurse would go, 'You know he's HIV positive.' I'm like, 'No.' So I went back and talked to him and I asked him had he ever gotten treated and he says, 'No.' I said, 'In 15 years you've never been treated and never been to the doctor?' And he said, 'No.' So, he actually came in the next day [be]cause I kept bugging him throughout the course of the evening while he was there. 'You're gonna come in tomorrow, right?' He actually got here at a quarter to eight in the morning and he called me from the ER, I was still at home and he says, 'I'm here.' And I was shocked because he was so intoxicated I didn't think he was gonna remember but I said, 'We have a lot of services, a lot of different programs and we can help you.' He showed up. So I was racing to get here, and brought him over, we fed him and got him talking to the social worker, you know, got him applied for Medi-Cal and got him into a shelter.'

#### The support of the medical setting

Finally, many program developers perceived that offering HIV testing was one way to demonstrate to ED patients they were receiving good medical care because every person should know his or her HIV status to stay healthy. They also viewed the medical environment as a significant benefit when disclosing a positive result because patients could feel that physicians were invested in their diagnosis and in ensuring they received the proper follow up care. One ED physician stated:

'I think that as awkward as it is to get a diagnosis of HIV this is the ultimate healthcare setting, right? This place is crawling with doctors and we've got specialists and you're here and we're gonna help you. And I think that as lonely as it might be getting that diagnosis you're also surrounded by - this is an environment that just bleeds medicine, right? I mean, I kind of think it's almost like an ideal situation.'

A testing program coordinator at another site echoed this sentiment:

'Because if a person's getting this diagnosis, it's pretty daunting. You know, you came in for a broken leg and you find out you have HIV. That person needs absolute support from all different directions medically and probably in some cases people are homeless or there are a lot of other issues around that diagnosis. And so what better way to support a person than when they're already there before you, and you can, while they're in the hospital, bring the services to them. You can make sure that the person has all the information they need about what it means to be HIV positive, that it's not a death sentence, it's a chronic illness like diabetes, there are things we can do to treat you.'

One tester pointed out that even though ED testing programs may emphasize and facilitate follow-up care, patients may perceive themselves as having other, more important health priorities, and that respecting patient autonomy was crucial, even if it meant acknowledging that patients might not want to be in HIV care:

'I believe the ED is where folks are coming for emergency care and so I think that whatever they're presenting with is what their issue is at that time. I think that HIV, even though it might be a very important diagnosis to me and everybody else up in there - you know what I mean - I think folks are still wanting to come in to be serviced for what they came in for, you know. And so I think that we should never forget that and that even though it's very important to us the HIV clinic should be another referral.'

## Discussion

In this assessment of the development and implementation of HIV testing in three EDs, we found distinct operational models - which we labelled the parallel model, the provider model, and the provider-parallel model - based on who offered and performed the test. The adoption and evolution of each model was shaped by local realities, including the relative contributions of ED and HIV physicians in creating the testing program, the criteria for patient selection, and the level of direct obligation on behalf of the testing program with regard to linkage to care. Similar to other studies that have described provider concerns over the provision of follow-up care [9,10], we found that the barrier to HIV testing common to all sites was concern over disclosure of a positive result. Our research also introduces several important facilitators that have been mentioned little, if at all, in the ED HIV testing literature: a commitment to caring for underserved populations; the additional yield of re-engaging known HIV-infected patients back into care; and the power of the medical setting in providing immediate support for a newly diagnosed HIV patient in the form of counselling, social services, and medical evaluation.

With regard to the barrier of discomfort about disclosing a positive HIV test result, some program staff felt that this concern arose in part from a stigmatized view of HIV and that more widespread HIV testing in the ED could help normalize perceptions of HIV among ED staff. One of the stated goals of the 2006 CDC guidelines was to reduce the stigma associated with HIV testing [1], and our findings highlight that this process has implications for healthcare providers as well as for patients. Based on our finding that the sites with existing programs were able to scale-up once ED staff developed a sense of familiarity with HIV testing, it is clear that comfort with the testing process is required before screening efforts can be maximized. Indeed, one study showed that ED residents experienced an increase in feelings of knowledge and confidence to conduct HIV counselling and testing after a six-month period of testing [10]. Thus, it may make sense for EDs interested in implementing HIV testing to consider beginning with pilot programs on a limited scale. Once a testing system has been shown to be feasible and acceptable to patients and providers, it can be refined and expanded. This conclusion is further supported by the fact all programs in this study worked by tailoring their use of outside funding, *i.e*., 'one size does not fit all.'

There were several limitations to our study. Our interviews were with 'key informants' who were active participants in efforts to monitor and improve program outcomes. A wider sampling of ED staff not directly involved in program management would likely have resulted in additional perspectives on barriers and facilitators of ED HIV testing. However, the stated purpose of this evaluation was to chronicle the histories of these programs while attending to barriers, facilitators, and factors influencing the adoption of testing process elements, not to evaluate programs in their larger contexts. This type of qualitative study would be an appropriate next step. We also recognize that these sites were participating in grant-funded projects, and thus may be more likely to champion the mission of ED HIV testing; however, we believe that the observations of this study are still be of use to other urban EDs considering HIV testing programs. In addition, as programs prefer to publish data on the uptake and yield of testing themselves, we do not provide information on the number of patients tested or testing positive. Finally, these qualitative data may not be generalizable across all ED HIV testing programs.

Several areas merit attention in future research. As emphasized in a 2009 Academic Emergency Medicine Consensus Conference on ED HIV testing [11], it is not clear how to sustain HIV testing in the ED in the absence of external funding and how to determine the optimal level of integration of HIV testing into ED activities. Further understanding the potential role of stigma on behalf of ED providers with regard to testing may help shed light on these important questions, since sustainable and integrated ED HIV testing will necessarily rely on attitudinal as well as financial support. Though one study has reported on attitudinal changes among ED residents before and after training and program implementation [10], more studies are necessary. Based on the variation in linkage to care practices among ED HIV testing programs, and the variation in where the responsibility for linkage to care lies, it is clear that we need to understand more about the process of entering care after an ED HIV diagnosis in order to optimize mechanisms for linkage to care.

## Conclusions

ED HIV testing can occur under a range of operational models that emerge from institutional strengths and are tailored to local realities. We identified three distinct models of HIV testing that vary along the spectrum of fully incorporating testing into the duties of existing staff to hiring additional staff to offer and perform the test. For all models, incremental program development may be a way to promote sustainable testing efforts. The combination of provider education and integrated linkage to care may help mitigate barriers around disclosure of a positive test result. Other staff feedback sessions can help enhance the key facilitators that emerged from this study: belief in the social mission of ED HIV testing, the perception that testing can connect out of care HIV patients to care, and the availability of social and medical resources in the ED to support patients newly diagnosed with HIV.

## Competing interests

The authors declare that they have no competing interests.

## Authors' contributions

SW, TL, JM, and SM conceived the study and obtained research funding. KC, KK, SW, and TL collected the data. KC, KK, and SW analyzed the data. KC drafted the manuscript and all authors contributed substantially to its revision. KC takes responsibility for the paper as a whole. All authors have read and approved the final manuscript.
